# Cardiovascular Risk and Modifiable Risk Factors in Shift-Working Healthcare Workers: A Gender-Stratified Cross-Sectional Study

**DOI:** 10.3390/jcm15114028

**Published:** 2026-05-22

**Authors:** Gabriele d’Ettorre, Gianmarco Giannelli, Francesco Branda, Giuseppe Loiacono, Gianluigi Calcagnile, Anna A. Centonze, Danilo Faggiano, Gabriella d’Ettorre, Giancarlo Ceccarelli

**Affiliations:** 1 Department of Occupational Medicine, Local Health Authority of Lecce, 73100 Lecce, Italygianluigimariafrancesco.calcagnile@asl.lecce.it (G.C.); annaadriana.centonze@asl.lecce.it (A.A.C.); danilo.faggiano@asl.lecce.it (D.F.); 2 Unit of Medical Statistics and Molecular Epidemiology, University Campus Bio-Medico of Rome, 00128 Rome, Italy; f.branda@unicampus.it; 3 Department of Public Health and Infectious Diseases, University of Rome Sapienza, 00185 Rome, Italy

**Keywords:** shift work, cardiovascular risk, healthcare workers, cross-sectional study, Italy

## Abstract

**Background**: Shift-working healthcare workers (HCWs) are at elevated cardiovascular (CV) risk due to chronic circadian disruption; however, gender-stratified data on CV risk profiles and modifiable risk factor distribution by occupational exposure duration remain scarce in the Italian hospital setting. This cross-sectional study aimed to characterise the 10-year CV risk profile and the distribution of modifiable risk factors in a hospital-based sample of shift-working HCWs. **Methods**: A retrospective cross-sectional study was conducted using data from routine occupational health surveillance of shift-working HCWs at a large Italian hospital in Salento, Southern Italy (survey year: 2025). The 10-year CV risk was estimated using the CUORE Project algorithm, validated for the Italian population. Risk was stratified by gender, age group, and shift work duration. Multivariable logistic regression models, adjusted for age, marital status, and presence of children at home, evaluated associations between selected risk factors and CV risk category. The study was reported in accordance with STROBE guidelines. **Results**: Of 765 HCWs included (320 males, 445 females; mean age 49.3 ± 8.5 years), male workers showed a significantly higher mean 10-year CV risk score (4.98 ± 2.8 vs. 1.34 ± 0.9; *p* < 0.05). Among male workers, the odds of moderate/high CV risk increased progressively with shift work duration (aOR 6.4 for >30 years). Males also showed significantly higher prevalence of arterial hypertension, overweight, and obesity across all strata. **Conclusions**: Male shift-working HCWs represent a higher-risk subgroup, characterised by a greater burden of modifiable cardiovascular risk factors. Integration of validated risk assessment tools into occupational health surveillance may support targeted preventive strategies in hospital settings.

## 1. Introduction

Cardiovascular disease (CVD) remains the leading cause of morbidity and mortality globally and represents a growing concern in occupational medicine. Among at-risk professional groups, shift-working healthcare workers (HCWs) are increasingly recognised as a population deserving targeted attention, given the dual burden of occupational stressors and lifestyle-related risk factors. Modern healthcare systems require continuous 24 h service provision, making rotating and night shift work an organisational necessity; however, the associated biological and behavioural consequences place workers at heightened cardiometabolic risk. Despite a substantial body of literature on shift work and health, data specifically characterising the cardiovascular risk profile of hospital-based HCWs using validated population-specific risk algorithms remain limited, particularly with regard to gender stratification and the role of modifiable risk factors across different exposure durations [1,2,3,4,5,6,7].

Circadian rhythms are endogenous, approximately 24 h cycles that regulate a wide range of physiological processes, including sleep–wake patterns, hormonal secretion, body temperature, blood pressure, and metabolic homeostasis. These rhythms are synchronized by environmental cues, primarily light exposure, but also by non-photic stimuli such as meal timing, physical activity, and social interactions [8]. Shift work, particularly night and rotating schedules, disrupts this synchronization process, resulting in circadian misalignment, defined as a mismatch between the endogenous biological clock and external environmental and behavioural cycles. Consequently, workers experience persistent desynchronisation, which may impair metabolic regulation and cardiovascular function, ultimately increasing the risk of cardiovascular disease [9].

Although the precise biological pathways linking shift work to adverse health outcomes are not yet fully elucidated, current scientific evidence supports two principal mechanisms: circadian misalignment and related neuroendocrine and metabolic alterations, including sleep disruption [1]. Chronic disruption of physiological rhythms can lead to neuroendocrine dysregulation, including alterations in cortisol secretion, increased sympathetic activity, and impaired glucose metabolism. Moreover, disturbances in hormonal and metabolic processes contribute to systemic inflammation, endothelial dysfunction, and a pro-atherogenic profile [10]. Over time, these alterations may promote the development of hypertension, insulin resistance, obesity, and dyslipidemia, which are well-established cardiovascular risk factors.

A substantial body of epidemiological research has demonstrated an association between shift work and cardiovascular diseases. Multiple systematic reviews and meta-analyses confirm an increased risk of coronary events, with a dose–response relationship related to the duration and intensity of shift work exposure [11,12]. In a recent prospective study, Skogstad et al. observed that rotating shift work, including night shifts, was associated with worsening cardiovascular risk profiles, including weight gain, increased arterial stiffness, assessed by carotid intima–media thickness and carotid–femoral pulse wave velocity, and elevated markers of systemic inflammation [13]. Consistently, Andreadi et al., in a systematic review, highlighted the link between shift work and cardiovascular diseases and proposed a mediating role of elevated cortisol levels, which may contribute to the development of hypertension, atherosclerosis, and other cardiovascular conditions [14].

Beyond biological mechanisms, behavioral and lifestyle factors may further amplify cardiovascular risk in shift workers. Irregular meal patterns, reduced physical activity, increased consumption of high-calorie foods, smoking, increased body mass index (BMI), and psychosocial stress are frequently observed among shift-working HCWs, potentially acting synergistically with circadian disruption to accelerate cardiometabolic risk [15,16,17,18]. Given this multifactorial scenario, increasing attention has been directed toward preventive strategies in occupational settings [19,20,21,22,23,24,25,26,27,28,29,30,31,32,33,34,35]. Targeted behavioral interventions aimed at modifiable risk factors, such as dietary counseling, promotion of physical activity, and stress management, have been proposed as potential measures to mitigate cardiovascular risk among shift workers [36,37,38,39,40,41,42,43,44,45,46,47,48,49,50,51,52,53,54,55].

Against this background, the rationale for the present study derives from the lack of gender-stratified cardiovascular risk data obtained using validated, population-calibrated tools within the specific occupational context of hospital shift workers in Southern Italy. Healthcare workers represent a particularly relevant target population: they are simultaneously exposed to the physiological burden of shift work and to the psychosocial stressors of high-demand clinical environments, yet paradoxically tend to underutilise preventive health services. Despite working within healthcare institutions, HCWs are not immune to preventable cardiovascular risk, and the occupational context may both amplify risk and create barriers to its recognition and management [56,57,58,59,60,61]. Specifically, while several high-quality international studies and meta-analyses have examined the association between shift work and cardiovascular risk in general working populations, including a large prospective UK Biobank cohort of 238,661 participants [62], a meta-analysis of 17 cohort studies comprising nearly one million participants [63], and a national Danish cohort of healthcare workers [64], none of these studies employed a risk algorithm calibrated for the Italian population, nor did they focus simultaneously on gender stratification and modifiable risk factor distribution across occupational exposure duration categories. The CUORE algorithm [24,25,27], as the only validated cardiovascular risk engine calibrated on Italian national epidemiological data, represents the methodologically appropriate tool for this purpose; yet its application within hospital-based occupational health surveillance remains absent from the published literature. This gap is particularly relevant because occupational health surveillance in Italy mandates periodic medical assessments for shift workers under Legislative Decree 81/2008; yet standardised cardiovascular risk profiling using population-specific tools is not universally implemented. This study addresses this gap through two objectives. The primary objective was to estimate the 10-year cardiovascular (CV) risk profile in shift-working HCWs undergoing routine occupational health surveillance, using the CUORE Project algorithm, the only cardiovascular risk tool validated for the Italian adult population. The secondary objective was to characterise the distribution of modifiable cardiovascular risk factors, specifically arterial hypertension, overweight, obesity, and physical inactivity, stratified by gender, age group, and duration of shift work exposure, to identify actionable targets for preventive strategies. Two design constraints must be noted from the outset: (1) no non-shift worker comparison group was included; (2) the study does not establish causal inference on the effect of shift work on cardiovascular risk. The findings are therefore descriptive and exploratory, intended to inform the design of surveillance-based prevention programmes.

## 2. Materials and Methods

### 2.1. Study Design and Setting

This retrospective observational cross-sectional study was conducted using data derived from the occupational health surveillance database of shift-working healthcare workers (HCWs) employed in a large hospital located in Salento, in the Southern region of Italy. The study included data collected between 2 January and 31 December 2025, during routine annual occupational health assessments performed by occupational physicians. The study was reported in accordance with the Strengthening the Reporting of Observational Studies in Epidemiology (STROBE) guidelines. A completed STROBE checklist is provided as Supplementary Material S1.

### 2.2. Study Population

The study population comprised all HCWs engaged in shift work and undergoing mandatory occupational health surveillance during the study period. Participants included medical doctors, nurses, laboratory technicians, and radiology technicians working under a forward-rotating shift schedule. The standard shift rotation at this institution consists of three shifts (morning: 07:00–14:00; afternoon: 14:00–21:00; night: 21:00–07:00), organised in a forward-rotating pattern (morning → afternoon → night). Workers typically rotate to the next shift type after a minimum of two consecutive days on the same shift, with a minimum inter-shift rest period of 11 h. Night shifts are performed on average 4–6 times per month, depending on ward requirements and professional category. Individual cumulative night shift counts were not available in the surveillance database for the full study period; years of shift work exposure was therefore used as a proxy measure of cumulative circadian disruption, acknowledging that this variable aggregates heterogeneous exposure intensities. Only workers with complete clinical and laboratory data required for cardiovascular risk estimation were included in the analysis, whereas non-shift workers and individuals with incomplete records were excluded.

### 2.3. Data Collection and Variables

Sociodemographic, clinical, and lifestyle data were systematically collected as part of routine occupational health evaluations. The recorded variables included age and gender, as well as anthropometric measures such as weight, height, and body mass index (BMI). Clinical parameters included systolic and diastolic blood pressure, while laboratory assessments comprised fasting glucose, total cholesterol, and high-density lipoprotein (HDL) cholesterol, as well as triglycerides. Lifestyle factors, including smoking status and physical activity levels, were also collected.

Physical inactivity was defined according to current international guidelines as engagement in less than 150 min per week of moderate-intensity activity or less than 75 min per week of vigorous-intensity activity [19,20]. Physical activity level was assessed through self-reported data collected during the occupational health interview by the occupational physician, using a standardised structured questionnaire included in the mandatory health surveillance protocol. The reliance on self-report is acknowledged as a potential source of measurement bias and is discussed in Section 4.2. Sleep quality data (e.g., validated questionnaire scores) were not systematically collected as part of the routine surveillance protocol and were therefore not available for inclusion in the analysis; this constitutes a recognised limitation of the study (see Section 4.2). Arterial hypertension was defined as systolic blood pressure ≥ 140 mmHg and/or diastolic blood pressure ≥ 90 mmHg [21,22,23].

### 2.4. Outcome Definition

The primary outcome of the study was the estimated 10-year cardiovascular (CV) risk. This was calculated using the validated algorithm developed within the CUORE Project, which is calibrated for the Italian population [24,25,26]. The algorithm integrates age, sex, systolic blood pressure, total cholesterol, HDL cholesterol, diabetes status, smoking habit, and antihypertensive treatment to estimate the risk of fatal and non-fatal coronary and cerebrovascular events over a 10-year period.

Based on the calculated score, participants were classified into three categories: low risk (<3%), moderate risk (≥3% and <20%), and high risk (≥20%) [27]. In addition, the distribution of individual cardiovascular risk factors, including arterial hypertension, overweight/obesity, smoking, and physical inactivity, was analyzed as secondary outcomes and stratified according to gender, age, and duration of shift work.

### 2.5. Statistical Analysis

Descriptive statistics were used to summarize the characteristics of the study population. Continuous variables were expressed as mean and standard deviation (SD), while categorical variables were reported as frequencies and percentages. Comparisons between groups were performed using the Mann–Whitney U test for non-normally distributed variables and Student’s *t*-test for normally distributed variables, whereas the chi-square (χ^2^) test was used for categorical data.

To assess the association between selected risk factors and the 10-year CV risk, multivariable logistic regression analyses were conducted separately for male and female workers, with results expressed as adjusted odds ratios (aORs) and 95% confidence intervals (CIs). Two separate models were constructed: (1) age-stratified analysis among male workers, with age group as the main predictor and CV risk category (moderate/high vs. low) as the dependent variable, adjusted for marital status and presence of children at home; (2) shift work duration analysis among male workers, with years of shift work as the main predictor and CV risk category as the dependent variable, adjusted for age, marital status, and presence of children at home. Female workers were not included in the regression models due to the negligible prevalence of moderate/high CV risk in this group across all strata, which precluded stable multivariable estimation. Physical inactivity was not included as a covariate in the regression models because its prevalence was uniformly high and did not differ significantly between male and female workers (72.6% vs. 71.9%, *p* = n.s.), thus precluding its use as a meaningful discriminating variable in gender-stratified analyses. Its potential role as a residual confounder is acknowledged in Section 4.2. A *p*-value < 0.05 was considered statistically significant. All statistical analyses were performed using SPSS software (Statistical Package for Social Sciences), version 14.0.

## 3. Results

**Figure 1:**
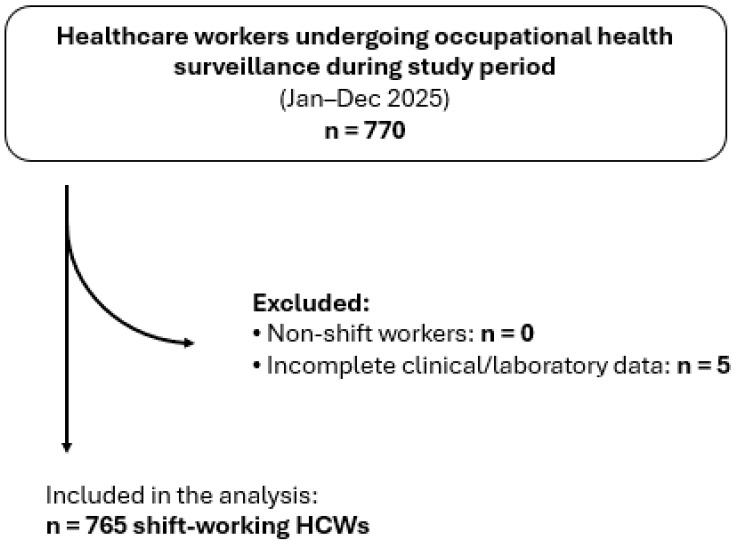
Study flow diagram.

A total of 765 shift-working healthcare workers (HCWs) were included in the study, of whom 320 were males and 445 females, with a mean age of 49.3 years (SD 8.5). The study selection process is illustrated in Figure 1. The source population of the occupational health surveillance database was already restricted to shift-working personnel at the time of data extraction; non-shift workers are managed under a separate surveillance pathway at the same institution and were not part of the assessment process from which data were drawn. Of the 770 records initially identified, 5 were excluded due to missing data required for CUORE algorithm calculation, resulting in a final analytic sample of 765 participants.

The main demographic and occupational characteristics of the study population are summarized in Table 1.

No statistically significant differences were observed between males and females in terms of age distribution, years of shift work, alcohol consumption, smoking habits, or levels of physical activity. However, significant gender differences emerged in anthropometric parameters. The prevalence of overweight and obesity was significantly higher among males compared to females (*p* < 0.05), whereas a higher proportion of females had a normal body weight.

With regard to the primary outcome, male shift-working HCWs exhibited a significantly higher 10-year cardiovascular (CV) risk compared to females. Before presenting the data, an important interpretive caveat must be stated: since sex and age are structural inputs of the CUORE algorithm, the finding that males show higher estimated CV risk than females, and that older workers show higher risk than younger ones, are partly inherent properties of the scoring formula rather than fully independent empirical observations. The primary contribution of this study lies in the characterisation of modifiable risk factors across occupational exposure strata, which are not structurally embedded in the algorithm; These are presented in detail in Table 1, Table 2, Table 3, Table 4 and Table 5 and discussed in Section Validity and Limitations of the CUORE Algorithm as an Outcome Measure in Occupational Research. Specifically, males were classified within the moderate-risk category, with a mean score of 4.98 (SD 2.8), while females showed a low-risk profile, with a mean score of 1.34 (SD 0.9) (*p* < 0.05). This difference is consistent with the structural properties of the CUORE algorithm and is presented here as a descriptive benchmark.

Further analyses were performed to explore the association between demographic and occupational variables and CV risk among male workers. It should be noted that the age-stratified analysis (Table 2) was included for descriptive completeness and to quantify the gradient of risk classification across occupational subgroups; however, given that age is a direct structural input of the CUORE algorithm, the observed association between age group and risk category is partially inherent to the algorithm’s construction rather than an independent empirical finding. This limitation is explicitly discussed in Section Validity and Limitations of the CUORE Algorithm as an Outcome Measure in Occupational Research. With this caveat, in stratified exploratory analyses, male shift workers in older age groups were significantly more likely to be classified in the moderate or high cardiovascular risk category. Compared with workers aged 35–45 years, the adjusted odds of being in the higher risk category were substantially greater in those aged 46–55 years (aOR: 3.57; 95% CI 1.8–7.1) and 56–69 years (aOR: 6.82; 95% CI 3.5–13.4), as shown in Table 2. Female workers were not included in the regression models, as the negligible prevalence of moderate or high CV risk across all female strata precluded stable multivariable estimation (see Section 2.5).

A consistent pattern was observed when stratifying by duration of shift work among male workers. Workers with longer occupational exposure were more likely to be classified in the moderate or high cardiovascular risk category, with significantly greater odds in those with 10–20, 21–30, and 31–40 years of shift work compared to those with less than 10 years (Table 3). Regression analyses for this outcome were restricted to male workers, as female participants consistently showed a low-risk CV profile (mean score 1.34 ± 0.9) with an extremely low prevalence of moderate or high CV risk across all shift work duration strata, making logistic regression modelling statistically uninformative in this subgroup. This dose–response pattern by exposure duration is of potential occupational health relevance, though the exploratory nature of the analysis and the structural overlap between predictors and the outcome score limit causal inference.

**Figure 2:**
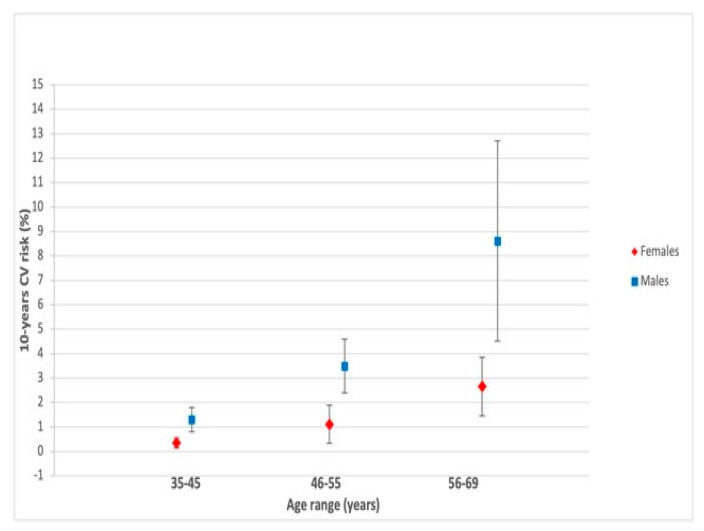
Estimated 10-year cardiovascular risk in shift-working HCWs stratified for age range. Y-axis: estimated 10-year cardiovascular risk (%). Error bars represent standard deviations (SD). Note: the CUORE algorithm uses age and sex as structural inputs; the pattern shown is partly a mathematical property of the scoring formula.

**Figure 3:**
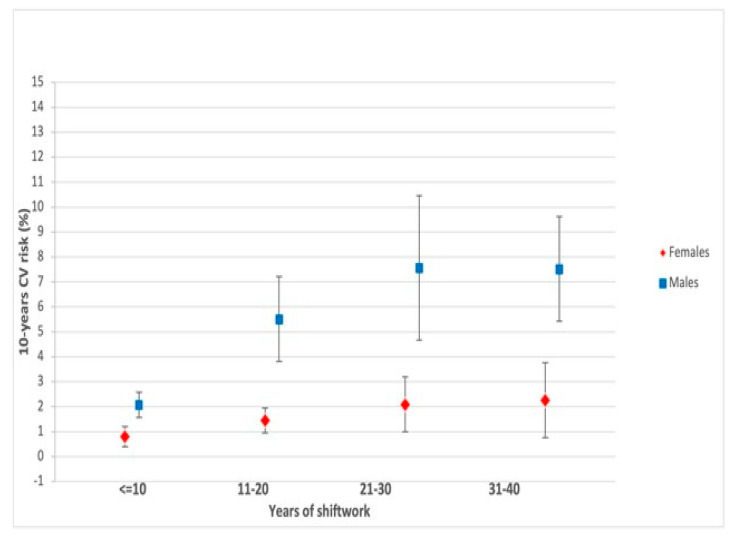
Estimated 10-year cardiovascular risk in shift-working HCWs stratified for years of shift work. Y-axis: estimated 10-year cardiovascular risk (%). Error bars represent standard deviations (SD). Note: years of shift work is used as a proxy for cumulative circadian disruption; individual night shift counts were not available.

These findings are consistent with the graphical representation shown in Figure 2 and Figure 3.

The distribution of individual cardiovascular risk factors further supported these findings. When stratified by age groups, male shift workers showed a significantly higher prevalence of arterial hypertension, overweight, and obesity compared to females across all age categories (Table 4).

Similarly, when stratified according to years of shift work, males consistently exhibited higher rates of hypertension, overweight, and obesity compared to their female counterparts (Table 5).

## 4. Discussion

In this retrospective observational study, we assessed the 10-year CV risk in a cohort of shift-working HCWs, stratified by gender, age, and duration of shift work exposure. The main findings of this study can be summarized as follows: (i) male shift workers exhibited a significantly higher 10-year CV risk compared to females; (ii) CV risk increased progressively with age and duration of shift work, particularly among males; and (iii) modifiable risk factors, including overweight/obesity, arterial hypertension, and physical inactivity, were more prevalent in male workers and were strongly associated with increased CV risk.

Our findings are consistent with well-established epidemiological evidence showing that cardiovascular morbidity and mortality are higher in males and increase with advancing age [16]. Although female HCWs exhibited a generally low 10-year CV risk across all age and length-of-service categories, a gradual increase was observed in older age groups, suggesting that the protective effect traditionally attributed to female sex may attenuate over time, possibly due to hormonal changes and cumulative exposure to cardiometabolic risk factors. In contrast, male shift workers showed a more pronounced risk profile, with significantly higher odds of moderate CV risk observed in workers aged >45 years and with more than 10 years of shift work exposure. This pattern suggests a potential cumulative effect of circadian disruption and lifestyle-related risk factors over time.

In our sample, the main variables associated with increased 10-year CV risk in males were overweight, obesity, arterial hypertension, and physical inactivity. These results are in line with the observational study by Tosoratto et al. [28], which reported increased CV risk predominantly among male shift workers and highlighted the mediating role of unhealthy lifestyle behaviors. In particular, smoking, physical inactivity, and unhealthy dietary patterns have been associated with atherogenic dyslipidemia and adverse lipid profiles, thereby contributing to elevated CV risk. Our findings support this interpretation and reinforce the hypothesis that behavioral factors may amplify the biological stress imposed by shift work.

Prospective evidence by Skogstad et al. [13] demonstrated that prolonged shift work is associated with early markers of arterial damage, including increased arterial stiffness and carotid intima–media thickness, which represent subclinical stages of atherosclerosis. Consistently, the meta-analysis by Madeira et al. [29] reported a significant association between shift work and increased blood pressure, with hypertension positively correlated with age and duration of exposure. These findings are in line with our observation of a higher prevalence of arterial hypertension among older male shift workers.

The relationship between increased BMI and arterial hypertension is well established [30,31,32]. Large population-based cohort studies have consistently demonstrated a strong and graded association between excess body weight and elevated blood pressure, with obesity recognised as one of the most important modifiable determinants of hypertension in working-age adults [30,31]. In this framework, Maniecka-Bryla et al. [33] specifically documented a high prevalence of hypertension among overweight and obese individuals in an occupational population, a finding directly relevant to the present context. Taken together, these findings support the role of obesity as a key intermediate factor in the pathway between prolonged shift work exposure and elevated cardiovascular risk, likely mediated through neuroendocrine dysregulation, increased sympathetic tone, and sodium retention associated with chronic circadian disruption [14,37]. The smoking prevalence observed in this cohort (26.2% in males, 20.9% in females) is relatively high for a healthcare workforce and exceeds national estimates for Italian HCWs, where smoking prevalence is generally reported in the 15–20% range. These elevated figures may partially reflect local sociocultural factors characteristic of Southern Italy, where tobacco use remains more prevalent than in Northern regions; however, the potential role of shift work itself as a determinant of smoking behaviour should not be overlooked. Evidence suggests that shift workers, particularly night shift workers, may be more likely to smoke than day-working counterparts, potentially as a coping mechanism for fatigue and circadian stress. Regardless of aetiology, the elevated smoking prevalence in this cohort represents a key modifiable cardiovascular risk factor that occupational health prevention programmes should explicitly address. The distribution of hypercholesterolaemia in this cohort also warrants brief comment: unlike other cardiometabolic variables, cholesterol abnormalities did not show a meaningful gender difference (Table 4 and Table 5). This contrasts with the broader pattern of male-predominant risk observed across the other factors and with general epidemiological data, which report higher LDL cholesterol in postmenopausal women relative to age-matched men. This finding may reflect the younger age profile of the female subgroup, pre-existing lipid-lowering treatment at assessment, or the limitations of binary categorical classification. It does not diminish the overall picture of male-predominant cardiometabolic risk in this cohort but highlights the importance of individualised risk factor evaluation beyond composite scoring.

According to converging evidence, the elevated CV risk observed in male shift-working HCWs may be driven by a combination of unhealthy lifestyle behaviors, high-strain occupational characteristics, and biological factors associated with circadian disruption [36,37]. Shift work disrupts normal daily routines, often leading to increased consumption of energy-dense foods and reduced physical activity, particularly during night shifts [38]. In this context, physical inactivity may act as a “risk multiplier,” exacerbating the metabolic consequences of circadian misalignment, including impaired glucose metabolism and increased insulin resistance [39]. The prevalence of physical inactivity in this cohort (~72% in both sexes) is notably higher than the 40–60% typically reported in European healthcare worker populations. This discrepancy warrants critical discussion. First, the single-item self-reported assessment used in routine occupational surveillance may systematically overestimate inactivity relative to accelerometry-based or multi-item questionnaire-based methods, and social desirability bias may further influence responses. Second, the high and homogeneous prevalence across genders limits the utility of physical inactivity as a discriminating variable in gender-stratified analyses, which is why it was not included as a covariate in the regression models (see Section 2.5). Third, the Southern Italian epidemiological context may genuinely reflect lower physical activity levels than Northern European comparator populations, consistent with existing data on regional variation in leisure-time physical activity across Italy. Future studies should adopt validated objective or multi-dimensional measures of physical activity to improve comparability.

In line with these findings, a large cross-sectional study involving over 53,000 workers demonstrated a significant association between shift work and increased prevalence of obesity and metabolic disorders, particularly among men [38]. Furthermore, physical activity is a well-established protective factor against cardiovascular disease, with evidence showing a 20–30% reduction in CV morbidity and mortality among physically active individuals. It exerts beneficial effects on lipid profile, blood pressure, glucose metabolism, and endothelial function. According to World Health Organization recommendations, regular physical activity remains a cornerstone of cardiovascular prevention [40].

Intervention studies further support these observations. Mamen et al. [41] demonstrated that targeted physical activity programs among rotating shift workers significantly improved blood pressure, glycemic control, and lipid profile. However, the Copenhagen General Population Study by Holtermann et al. [42] highlighted the “physical activity paradox,” showing that while leisure-time physical activity is protective, high levels of occupational physical activity may be associated with increased cardiovascular risk.

Gender differences in CV risk may also be partially explained by differences in lipid metabolism. Emerging evidence suggests that circadian disruption may induce a specific dyslipidemic profile in shift workers, characterized by hypertriglyceridemia, reduced HDL cholesterol, and increased levels of atherogenic lipoproteins [43,44,45,46]. These alterations may contribute to the higher CV risk observed in male shift workers.

Consistently, Zhou et al. [47] reported sex-specific differences in cardiac function among shift workers, with male workers showing impaired left ventricular function compared to females. However, contrasting evidence has been reported by Angeli et al. [48], who highlighted an increased susceptibility to type 2 myocardial infarction in females, likely related to hormonal and microvascular factors. These findings underline the complexity of gender-related differences in cardiovascular risk.

### 4.1. Clinical and Occupational Health Implications

Our findings have relevant implications for both clinical practice and occupational health policies. The identification of male shift-working HCWs as a higher-risk subgroup highlights the need for targeted preventive strategies focusing on modifiable risk factors. Interventions aimed at weight management, promotion of physical activity, and improvement of dietary habits may substantially reduce long-term cardiovascular risk.

Furthermore, the progressive increase in CV risk with longer exposure to shift work suggests the importance of early risk stratification and periodic cardiovascular monitoring in this population. Occupational health surveillance programs should integrate validated CV risk assessment tools, such as the CUORE algorithm, to identify high-risk individuals and implement timely preventive interventions.

From an organizational perspective, optimizing shift schedules and implementing ergonomic scheduling models may further mitigate the impact of shift work on cardiovascular health. Strategies including adequate rest periods between shifts, forward-rotating schedules, and worker participation in shift planning have been associated with improved work–life balance and reduced physiological stress [56,57,62]. In addition, workplace-based health promotion programs focusing on sleep hygiene, physical activity, and nutrition may represent effective approaches to reduce cardiometabolic risk among shift workers [49,50,51,52]. It should be noted, however, that the present findings derive from a single-centre sample of shift workers with no comparator group; accordingly, the clinical and organisational implications outlined above should be interpreted as hypothesis-generating rather than definitive, and their applicability to other settings requires validation in larger, multi-centre studies. A further sex-specific consideration of clinical relevance concerns the potential role of obstructive sleep apnea syndrome (OSAS) as an under-recognised cardiovascular risk factor in shift-working women. Although a systematic review and meta-analysis on the association between shift work and possible OSAS found the evidence inconclusive (RR = 1.05; 95% CI 0.85–1.30) [65], the circadian disruption inherent to rotating and night shift schedules may predispose shift workers to sleep-disordered breathing through fragmented sleep architecture and altered upper airway tone. Importantly, while OSAS is generally more prevalent in men, its prevalence increases sharply in women after menopause, a pattern that may partially explain the attenuation of the female protective effect against cardiovascular disease observed in older age groups in the present study. Postmenopausal women with OSAS exhibit a substantially higher burden of cardiometabolic risk factors, including hypertension, dyslipidaemia, and impaired glucose tolerance, all of which are structurally relevant to the CUORE algorithm inputs [57]. These considerations reinforce the importance of sex-specific screening strategies within occupational health surveillance programmes, including systematic assessment of sleep quality and sleep-disordered breathing risk in shift-working women approaching or beyond the menopausal transition.

#### Validity and Limitations of the CUORE Algorithm as an Outcome Measure in Occupational Research

The use of the CUORE algorithm as the primary outcome measure in this study requires critical appraisal from both a methodological and a conceptual perspective. The algorithm was developed and validated on a representative sample of the Italian adult general population and integrates age, sex, systolic blood pressure, total and HDL cholesterol, diabetes, smoking habit, and antihypertensive treatment as input variables [24,25,26,27]. Its calibration for the Italian epidemiological context constitutes a key methodological strength of the present study, enabling population-specific risk estimation that alternative international algorithms, such as the Framingham Risk Score or SCORE2, would not provide with equivalent precision for this setting. However, a critical interpretive limitation arises from the structural embedding of age and sex within the algorithm: the finding that male workers carry a higher estimated 10-year CV risk than females, and that risk increases progressively with age, is, to a substantial degree, a mathematical consequence of the CUORE formula rather than a purely empirical observation. These findings therefore lack independent novelty and must be interpreted strictly within this constraint. This limitation is explicitly acknowledged in Section 3 and in the design constraints stated in the Introduction.

The primary added value of the present study lies in the stratified characterisation of modifiable cardiovascular risk factors, specifically arterial hypertension, overweight, obesity, and physical inactivity, across categories of shift work duration (Table 4 and Table 5). These variables are not structurally embedded in the CUORE algorithm and therefore constitute independent findings with direct relevance for occupational health programme design. In particular, the progressive increase in the prevalence of hypertension, overweight, and obesity with increasing years of shift work among male workers aligns with biological hypotheses linking cumulative circadian disruption to cardiometabolic dysregulation [13,14,37], and provides empirical grounding for exposure-duration-based risk stratification in occupational surveillance.

The multivariable analyses presented in Table 2 and Table 3 should be interpreted as exploratory stratification models, designed to quantify the adjusted probability of belonging to the moderate or high cardiovascular risk category across occupational subgroups, defined by age stratum and shift work duration category, rather than as models intended to explain or decompose the CUORE score. A structural collinearity exists between age and the outcome, since age is a direct input to the CUORE algorithm; this limits the capacity of age-stratified regression models to isolate the independent contribution of occupational exposure duration from that of biological ageing per se. Accordingly, the dose–response pattern observed across shift work duration strata must be interpreted with caution and should not be construed as establishing a causal pathway. These limitations are further addressed in Section 4.2.

### 4.2. Limitations

This study has several limitations that should be considered when interpreting the findings. First, the single-centre design and the restriction to shift-working personnel from a single hospital in Southern Italy may limit the generalisability of the results to other healthcare settings, geographical contexts, or professional categories not represented in the sample. Second, the retrospective cross-sectional design precludes prospective follow-up for incident cardiovascular events; accordingly, the analysis is based on estimated 10-year CV risk rather than observed clinical outcomes, and causal inference cannot be established. Third, the CUORE Project risk algorithm, although validated for the Italian general adult population, was not specifically developed for occupational subpopulations engaged in shift work and may not fully capture the additional cardiometabolic risk attributable to chronic circadian disruption; this limitation has been explicitly addressed in Section Validity and Limitations of the CUORE Algorithm as an Outcome Measure in Occupational Research. Fourth, several potentially relevant confounders were not available for inclusion in the analysis, including objective measures of sleep quality, dietary patterns, psychosocial work demands, and specific shift schedule characteristics (e.g., number of consecutive night shifts, speed of rotation). Fifth, physical inactivity, although highly prevalent in both genders, was not incorporated as a covariate in the regression models due to the absence of a significant gender difference in its prevalence; its potential role as a residual confounder in within-group analyses should be considered when interpreting the regression findings. Sixth, the statistical analysis was conducted using SPSS version 14.0, which represents an older software release; while results are not expected to differ substantially from those obtained with more recent versions, future studies should adopt updated analytical platforms. Finally, some references cited in support of background statements and discussion points were published more than five years prior to submission; where possible, these have been replaced by more recent evidence; however, foundational references related to the CUORE algorithm validation methodology [24,25,27] and certain epidemiological findings without more recent equivalents have been retained and explicitly justified. An additional and important limitation concerns the potential confounding of age in the shift work duration analysis. Workers with longer occupational exposure are necessarily older, and since age is a direct input of the CUORE algorithm, the apparent dose–response pattern between years of shift work and CV risk category is substantially influenced by this structural relationship. Although the regression models were adjusted for age, residual confounding cannot be excluded. A sensitivity analysis restricted to workers aged under 55 years, which would attenuate the age–exposure overlap, was not feasible in the current dataset due to the resulting reduction in sample size; this represents a priority for future, larger data collection.

## 5. Conclusions

This cross-sectional, surveillance-based study characterises the 10-year cardiovascular risk profile and the distribution of modifiable risk factors in a cohort of shift-working healthcare workers, with a focus on gender differences and occupational exposure duration. Male shift-working HCWs showed a significantly higher estimated 10-year cardiovascular risk compared to females, a pattern that is partly consistent with the structural properties of the CUORE algorithm but is independently supported by a greater burden of modifiable risk factors, i.e., high blood pressure, overweight, obesity, and physical inactivity, across shift work duration categories. The progressive increase in the prevalence of these factors with longer occupational exposure among male workers suggests that duration of shift work may serve as a useful criterion for prioritising preventive cardiovascular interventions within occupational health surveillance frameworks. These findings support the routine integration of validated, population-calibrated cardiovascular risk assessment tools, such as the CUORE algorithm, into occupational health surveillance programmes in hospital settings. Structured preventive programmes targeting lifestyle-related risk factors, including weight management, promotion of physical activity, blood pressure control, and assessment of sleep-disordered breathing in postmenopausal women, may be of particular value for HCWs with prolonged shift work exposure. Future longitudinal studies incorporating non-shift worker comparison groups, objective measures of circadian disruption, and validated sleep quality assessments are needed to establish causal pathways and evaluate long-term preventive effectiveness in this population.

## Figures and Tables

**Table 1:** Sample demographics.

	**N.**	
Entire group	765	
Females	445	
**Age range (years)**	**Females N (%)**	**Males N (%)**
35–45	129 (29%)	97 (30.3%)
46–55	165 (37.1%)	109 (34.1%)
56–69	151 (33.9%)	114 (35.6%)
**Job**	**Females N (%)**	**Males N (%)**
Medical Doctors	104 (23.4)	91 (28.4)
Nurses	215 (48.3)	129 (40.3)
Technical Staff	44 (9.9)	38 (11.9)
Healthcare Assistant	82 (18.4)	62 (19.4)
**Years of shiftwork**	**Females N (%)**	**Males N (%)**
<10	146 (32.8%)	120 (37.5%)
11–20	174 (39.1%)	116 (36.2%)
21–30	101 (22.7%)	68 (21.3%)
30–40	24 (5.4%)	16 (5%)
**Smoking habit**	**Females N (%)**	**Males N (%)**
	93 (20.9)	84 (26.2)
**Alcohol consumption**	**Females N (%)**	**Males N (%)**
	139 (31.2)	108 (33.7)
**Physical inactivity**	**Females N (%)**	**Males N (%)**
	320 (71.9%)	232 (72.6)
**10-year CV risk**	**Females (SD)**	**Males (SD)**
	1.34 (0.9)	4.98 (2.8)
**BMI**	**Females N (%)**	**Males N (%)**
<18.5	13 (2.9)	7 (2.2)
18.5–24.9	266 (59.8)	131 (41)
25–29.9	129 (29)	141 (44.1)
≥30	37 (7.6)	41 (12.8)

**Acronyms**: CV = cardiovascular, HCW = healthcare worker, BMI = body mass index.

**Table 2:** Exploratory logistic regression analysis (CV risk category as surrogate outcome) among male shift-working HCWs, stratified by age.

Age Range	OR (95%CI) *	*p* Value
35–45	1 **	
46–55	3.57 (1.8–7.1)	<0.001
55–69	6.82 (3.5–13.4)	<0.001

* Exploratory logistic regression. Dependent variable: CV risk category (moderate/high vs. low), derived from CUORE algorithm. Note: age is a structural input of CUORE; these models should be interpreted as descriptive stratification analyses, not as evidence of independent causal associations. Adjusted for marital status and dependent children. ** referent category. **Acronyms**: aOR = adjusted odds ratio, CI = confidence interval.

**Table 3:** Exploratory logistic regression analysis (CV risk category as surrogate outcome) among male shift-working HCWs, stratified by years of shift work.

Years of Shiftwork	OR (95%CI) *	*p* Value
<10	1 **	
10–20	3.9 (2.1–7.1)	<0.001
21–30	4.2 (2.1–8.2)	<0.001
31–40	6.4 (2.1–19.2)	<0.001

* Exploratory logistic regression. Dependent variable: CV risk category (moderate/high vs. low), derived from CUORE algorithm. Note: age is a structural input of CUORE; these models should be interpreted as descriptive stratification analyses, not as evidence of independent causal associations. Adjusted for marital status and dependent children. ** referent category. **Acronyms**: aOR = adjusted odds ratio, CI = confidence interval.

**Table 4:** Distribution of modifiable cardiovascular risk factors in shift-working HCWs, stratified by age group. * *p* < 0.05 compared to female shift-working HCWs.

Age-Range (Years)	Arterial Hypertension (%)		Hypercholesterolemia (%)		Diabetes (%)		Overweight (%)		Obesity (%)	
	M	F	M	F	M	F	M	F	M	F
35–45	11.3 *	8.5	22.7	23.3	2.1	2.3	38.1 *	27.1	7.2 *	3.9
46–55	31.2 *	21.2	28.4	29.1	2.8	3.6	44.1 *	31.6	9.2 *	5.4
56–69	50.9 *	35.1	34.2	35.8	6.1	6.6	58.8 *	37.1	17.5 *	12.6

**Table 5:** Distribution of modifiable cardiovascular risk factors in shift-working HCWs, stratified by duration of shift work exposure (years). * *p* < 0.05 compared to female shift-working HCWs. Values expressed as mean (SD) for continuous variables and % (n) for categorical variables.

Years of Shiftwork	Arterial Hypertension (%)		Hypercholesterolemia (%)		Diabetes (%)		Overweight (%)		Obesity (%)	
	M	F	M	F	M	F	M	F	M	F
<10	6.7 *	5.5	19.2	20.6	2.5	(4) 2.7	36.7 *	26.1	6.7 *	3.4
10–20	12.1 *	8.1	21.6	23.6	2.6	(5) 2.9	43.1 *	29.9	8.6 *	5.2
21–30	29.4 *	19.8	33.8	36.6	5.9	(7) 6.9	57.4 *	36.6	16.2 *	10.0
31–40	56.2 *	25.1	37.5	37.5	6.2	(2) 8.3 *	56.2	37.5	18.7 *	8.4

No consistent gender differences were observed for hypercholesterolemia or diabetes.

## Data Availability

Data are available upon request.

## Supplementary Materials

The following supporting information can be downloaded at: https://www.mdpi.com/article/10.3390/jcm15114028/s1, Supplementary Material S1—STROBE checklist.

## Abbreviations

The following abbreviations are used in this manuscript:

HCW	Healthcare worker
CV	Cardiovascular
CVD	Cardiovascular disease
BMI	Body mass index
LDL	Low-density lipoprotein
HDL	High-density lipoprotein
