# Supplementary material for: Cardiovascular Risk and Modifiable Risk Factors in Shift-Working Healthcare Workers: A Gender-Stratified Cross-Sectional Study
Source: J Clin Med. 2026 May 22;15(11):4028. doi: 10.3390/jcm15114028 (PMC13258224; doi:10.3390/jcm15114028)
Supplement: Supplementary file 1 [file jcm-15-04028-s001.zip › jcm-4286038-supplementary.pdf]

Supplementary Material S1 – **STROBE checklist**

Study: Cardiovascular Risk in Shift-Working Healthcare Workers: A Gender-Based Analysis from a Retrospective Hospital-Based Study

Item	Recommendation	Reported	Location	Page
**1**	Study design in title/abstract	Yes	Title; Abstract (Methods)	p.1
**2**	Informative abstract	Yes	Abstract	p.1
**3**	Background/rationale	Yes	Introduction (paragraphs 1–5)	pp.2–3
**4**	Objectives clearly stated	Yes	Introduction (final paragraph)	p.4
**5**	Study design	Yes	Section 2.1 Study Design and Setting	p.4
**6**	Setting	Yes	Section 2.1 Study Design and Setting	p.4
**7**	Participants	Yes	Section 2.2 Study Population	p.5
**8**	Variables	Yes	Sections 2.3 Data Collection and Variables; 2.4 Outcome Definition	pp.5–6
**9**	Data sources/measurement	Yes	Section 2.3 Data Collection and Variables	p.5
**10**	Bias	Yes	Section 4.2 Limitations	p.13
**11**	Study size	Yes	Section 4.2 Limitations	p.13
**12**	Quantitative variables	Yes	Section 2.5 Statistical Analysis	p.6
**13**	Statistical methods	Yes	Section 2.5 Statistical Analysis	p.6
**13a**	Control for confounding	Yes	Section 2.5 Statistical Analysis	p.6
**13b**	Subgroups/interactions	Yes	Section 3 Results (stratification by gender, age, shift work duration)	pp.7–10
**13c**	Missing data	Yes	Section 2.2 Study Population (exclusion of incomplete records)	p.5
**13d**	Sensitivity analyses	No	Not reported	—
**14**	Participants flow	Yes	Section 3 Results (opening paragraph)	p.7
**14a**	Non-participation reasons	Yes	Section 2.2 Study Population	p.5
**14b**	Flow diagram	Yes	Figure 1	p.7
**15**	Descriptive data	Yes	Table 1 + narrative description	pp.7–8
**16**	Outcome data	Yes	Section 3 Results + Tables 2–3	pp.8–10
**17**	Main results (estimates, CI)	Yes	Tables 2–3	p.9
**18**	Other analyses	Yes	Tables 4–5	pp.10–11

**19**	Key results	Yes	Section 4 Discussion (opening paragraph)	p.11
**20**	Limitations	Yes	Section 4.2 Limitations	p.13
**21**	Interpretation	Yes	Section 4 Discussion	pp.11–12
**22**	Generalisability	Yes	Section 4.2 Limitations	p.13
**23**	Funding	Yes	Funding section	p.14
